# A quality of life index for the rural periphery of Sri Lanka using GIS multi-criteria decision analysis techniques

**DOI:** 10.1371/journal.pone.0308077

**Published:** 2024-09-18

**Authors:** Neel Chaminda Withanage, Kalpani Lakmali Gunathilaka, Prabuddh Kumar Mishra, Kamal Abdelrahman, Dilnu Chanuwan Wijesinghe, Vishal Mishra, Sumita Tripathi, Mohammed S. Fnais

**Affiliations:** 1 Faculty of Humanities and Social Sciences, Department of Geography, University of Ruhuna, Matara, Sri Lanka; 2 School of Geographical Sciences, Southwest University, Bebei District, Chongqing, P. R. China; 3 Faculty of Arts, Department of Geography, University of Colombo, Colombo, Sri Lanka; 4 Department of Geography, Shivaji College, University of Delhi, New Delhi, India; 5 Department of Geology and Geophysics, College of Science, King Saud University, Riyadh, Saudi Arabia; 6 Helmholtz Centre Potsdam, Remote Sensing and Geoinformatics section, GFZ German Research Centre for Geosciences, Telegrafenberg, Potsdam, Germany; 7 Department of Environment Studies, Shri Lal Bahadur Shastri National Sanskrit University, New Delhi, India; COMSATS University Islamabad - Lahore Campus, PAKISTAN

## Abstract

Spatial evaluation of the region is associated with the assessment of the Quality of Life (QoL). Despite numerous research endeavoring to define, measure, quantify, and map the quality of life, there exists a consistent fault in Sri Lanka. Hence, the objective of this study was to construct a QoL index and determine the spatial disparities of QoL from the Polpitigma town to its periphery. The assessment was conducted by employing 20 geographical factors that quantify QoL using the Geographic Information Systems (GIS). The evaluation assigned weights to each criterion based on the assessments of both local residents and experts, utilizing the Multi-Criteria Decision Analysis (MCDA) and the Analytical Hierarchy Process (AHP). The findings indicated that cultural factors made a greater contribution compared to the environment,service functions,security and socioeconomic factors. Within the study area, the region with a higher quality of life (HQoL) only covered 4.5% (17.3 km^2^), whilst the lower QoL zone encompassed 63.8% (252 km^2^). And also, the distance from the town is a crucial factor in determining the spatial variations in QoL. The derived model can serve as a road map for local-level planning, as it has been validated and shown to have an accuracy of 74% through the Receiver operating characteristic (ROC) curve. Considering the lack of previous research in this field, this study offers a crucial contribution in enhancing the QoL for underprivileged communities in the study area by improving employment, income, and accessibility to physical infrastructure, public utility services, and cultural and recreational facilities. Especially the findings of this study can efficiently guide decisions for the distribution of financial resources to enhance the QoL in impoverished rural communities on the rural periphery of DS.

## Introduction

The topics of Quality of Life (QoL) and social well-being have long been the focus of attention. However, in recent decades, there has been a rise in studies exploring these subjects using multidisciplinary approaches. Researchers in the fields of social science, environmental science, and health science have shown a special interest in topics related to the quality of life [1]. Consequently, numerous definitions of QoL have been published in the international literature [2]. Contrary to the commonly held idea that QoL is mostly linked to health, in actuality QoL is a complex subject that poses difficulties in its definition from a singular standpoint. Various factors were considered when addressing QoL, as indicated (**S1 Fig**) in previous research [3,4]. Thus, it is evident that QoL is centred around enhancing one’s overall well-being, as explicitly outlined by the World Health Organization’s Quality of Life (WHOQOL)[5]. The World Health Organization (2012) defines quality of life as the subjective evaluation individuals make about their position in life, taking into account their objectives, ambitions, standards, and concerns, within the framework of the culture and value systems of their society. This method encompasses various components like as physical well-being, psychological state, degree of autonomy, social relationships, and personal beliefs, along with their interactions with significant environmental elements [6].The research remains focused on assessing the quality of life, taking into account the physical and social environment, societal expectations, and the changing importance of these needs throughout time. Several recent studies have been conducted to evaluate the QoL on both a local and global scale, and several methodologies have been proposed for these assessments. QoL indices and maps facilitate decision-making for targeted interventions aimed at enhancing QoL within the focused area by effectively displaying the spatial distribution [7–10]. Extensive study has consistently shown that an individual’s QoL is closely linked to their residential location. In simpler terms, a favourable living environment is indicative of a favourable life [11–13]. Therefore, when assessing the QoL in a certain area, it is crucial to primarily consider the characteristics that contribute to the overall QoL in that particular place. Prior studies have shown that a diverse set of indicators and criteria, encompassing demographic, socioeconomic, and physical characteristics, can be employed to evaluate the quality of life in different geographical areas. Examining the spatial analysis of the QoL of individuals in different regions is particularly advantageous and essential for decision-makers in local-level governance, as it effectively illustrates the distribution of resources in the area. Thus, much research has been conducted to guide government agencies and policymakers in an international context though it is very rare in Sri Lanka.

According to the expert’s perspective, the optimal and suitable conclusions are often achieved through the evaluation of numerous possibilities, a process aided by the application of the AHP. MCDA approaches were created by two schools: American and European operational research schools. While the American School largely concentrate on the functional approach that leverages value, the European school focuses on the relational notion. In this context, the AHP, TOPSIS, and MAUT methodologies are the most often used MCDA approaches created by American schools. ELECTRE, PROMETHEE, and NAIADE are the most prominent MCDA techniques established by European schools. Because AHP can recognize and balance the relevance of complex aspects, researchers regularly utilize it to support decision-making in the process of environmental planning and natural resource management. Over mentioned MCDM methodologies, AHP obtains more attention in suitability analysis due of its flexibility and practical application. Thus, in many prior studies, most researchers have utilized AHP to locate suitable sites in diverse spatial and socioeconomic perspectives. AHP can calculate the ratio values for many criteria by means of pairwise comparison. Subsequently, weights can be allocated to each criterion for assessment [14]. The AHP is a highly effective technique for policy-making that involves generating ratio scales from the collection of judgement [15–19]. During the AHP process, several processes must be undertaken, including establishing objectives, delineating criteria and elements for various levels, and ultimately constructing a hierarchical structure [20–22]. The AHP and MCDA are extensively employed in spatial decision-making research because to their numerous benefits, such as time efficiency and cost-effectiveness. MCDA can be employed as a methodology to minimize cost and time in spatial decision-making. Therefore, it is evident that GIS is a crucial tool for accurately identifying the connections between several criteria [20,23].

A number of studies have been conducted in the context of life quality variance employing GIS technology. Faka et al., [24] used a variety of QoL influencing characteristics to customize and evaluate life quality in Greece. Another study suggests an integrated methodology for analyzing and mapping QoL at a micro-scale in Katerini, Greece [6]. Studies have also been conducted by integrating GIS and location-allocation models with MCDA. El Karim and Awawdeh [25] attempted to assess the QoL in Buraidah City, Saudi Arabia. Zhong et al., [26] used a data-driven analytical strategy to examine living handiness in Kaifeng City, China using multi-sourced urban information and geo-design methods on an individual scale. Merschdorf et al., [27] attempted to customize and analyze the correlation between urban attributes and peoples’ discerned quality of city life using statistical analysis and geospatial analysis. Karadimitriou et al., [28] analyzed the spatial distribution of multiple deprivations and established a connection between geographical patterns and the history of urban development access in Athens. Garau and Pavan [29] have demonstrated that the development of qualitative and quantitative descriptors of urban environments can benefit from a system of indicators. By combining census data warehouse analysis with remote sensing-derived characteristics, Rao et al., [30] in their study also evaluated the quality of life in the state of Uttarakhand, India utilizing Geoinformatics. All the studies revealed that life quality mapping is a powerful decision-making tool that identifies the factors to be considered to improve life quality in a particular area. However, due to Sri Lanka’s lack of technical advancement, it is difficult to access research that aids decision-makers, particularly in local-level planning. There is only one available research conducted by Dissanayake et al.,[23], which discusses the evaluation of QoL using the GIS technique. This research specifically focuses on the city of Kandy in Sri Lanka.This study focuses on the development of a QoL index. The index is constructed based on 13 criteria and highlights the application of GIS to visually represent spatial variations in QoL.

Galagamuwa, Mahawa, Polpitigama, and Abanpola DS in the Kurunegala district are economically underdeveloped because of the geographical and socioeconomic disparities. Polpitigama is particularly notable due to human-elephant conflicts and droughts as well.Therefore, rural communities in Polpitigama are encountering numerous challenges in their day-to-day living. Hence, it is crucial to implement remedial planning measures at the local level in order to enhance the QoL for the underprivileged segment. Thus, this DS was chosen as the experimental object. Building upon the same limitation observed in earlier research conducted at both national and local levels, this study aimed to derive a QoL index and evaluate the spatial disparities of QoL using spatial techniques within a relatively small geographical area. This approach was chosen to ensure more accurate and reliable results, since it effectively accounts for spatial heterogeneity.Therefore, this study tried to address the current deficiencies in research by incorporating specific QoL factors and indicators that are relevant to the underdeveloped study area.Hence, a QoL variation index was created in Polpitigama DS by incorporating various variables in the GIS environment. Additionally, an attempt was made to quantify the impact of the distance from the city centre on differences in QoL as one moves out from the town center. The reliability of the derived index was ensured by validating the QoL map through field verification. This will serves as a comprehensive road map for local-level planning in the study area, with the goal of improving the QoL.

## Materials and methods

### Description of the study area

**Fig 1:**
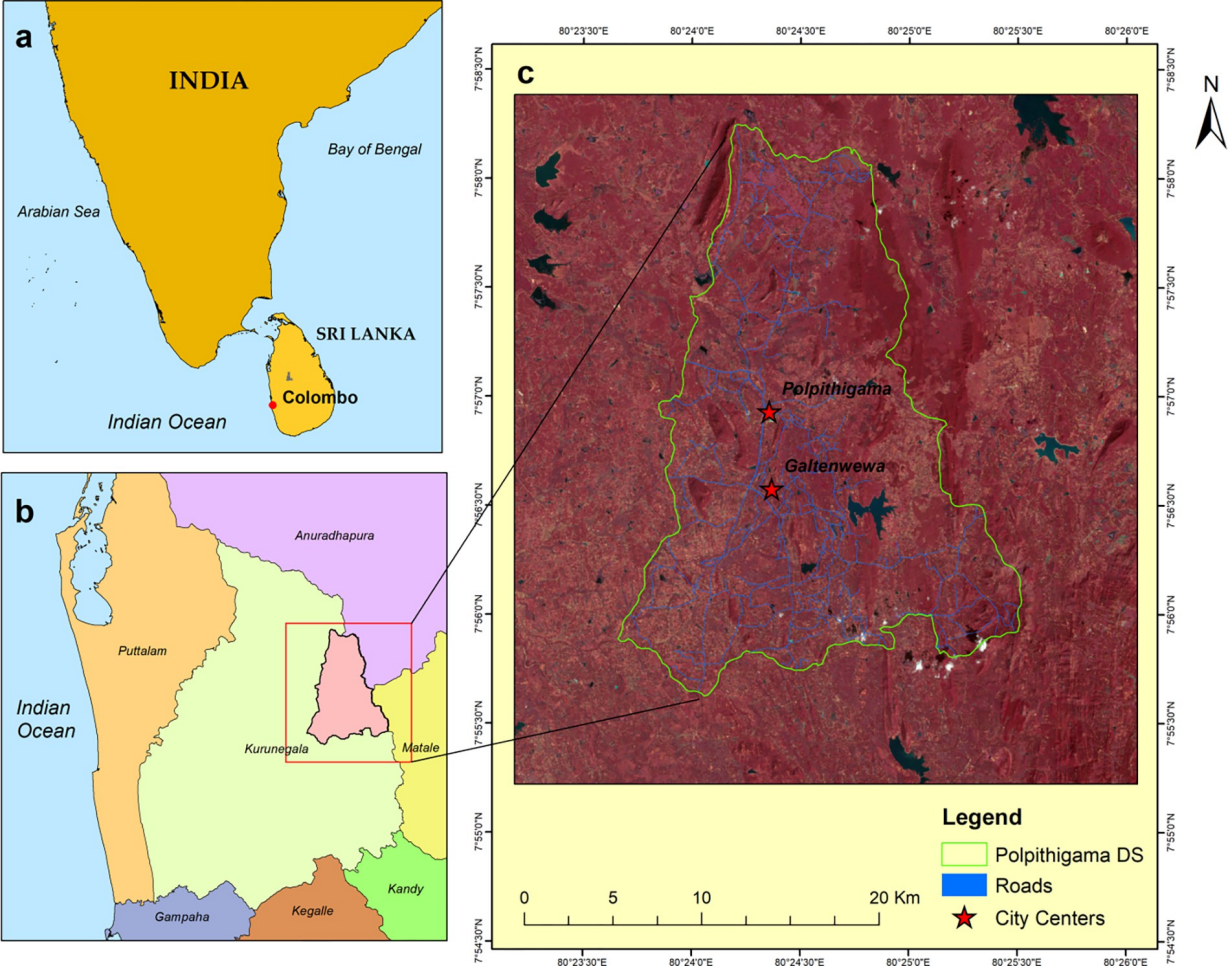
Location maps: **(a)** Sri Lanka in the Indian Ocean.The World topographic map was obtained at ArcGIS and is the property of ESRI, used herein under license [31]. The copyrights belong to ESRI, but according to the terms of use, the copyright holder does not need to apply for permission to use because it is free for academic publications, and can be used freely and commercially under the CC BY 4.0 license.; **(b)** Location of Polpitigama DS.; **(c)** Polpitigama DS in Landsat8 false colour (5,4,3) composite. Map was edited by authors using United States Geological Survey Earth Explorer Landsat images [32].

The Polpitigama Divisional Secretariat is situated in the Kurunegala District (**Fig 1**) of the North Western Province in Sri Lanka. It is positioned between 80°.20 E to 80°.32 E’ and 7°.40’ N to 7°.79’ N latitude, covering a land area of 389.9 km^2^. The research area is delimited by the Divisional Secretariats of Glanewa and Palagala to the north, Galewela and Ibbagamuwa to the east, Ganewathha DS to the south, and Mahawa and Ehetuwewa to the west, based on its relative location. The DS elevation varies from 77m and 506m above mean sea level. The DS exhibits a combination of dry and intermediate climatic characteristics, with an approximate mean annual temperature of 23.4–C. Based on the US Air Quality Index (AQI) value, it is evident that the air quality in the Kurunegala district, including the study region, is within the usual range, typically ranging from 28 to 37. The southern part of the DS generally experiences an annual precipitation of 1750 mm, whereas the northern area normally receives 1500 mm. The study area consisted of 294 villages and 82 Grama Niladhari Divisions, with 93,795 total population, covering a land area of 38,995 hectares. Among the 22,994 household units, 16,946 (41.2%) are engaged in agricultural activities, while 5,684 individuals work in government sectors and 364 in semi-government sectors. The total number of dwellings is 25,682, consisting of 21,926 permanent residences, 2,999 semi-permanent homes, and 757 temporary residences.

### Materials

The study utilized 20 spatial data layers, which used in earlier research on GIS-integrated QoL assessment and formal interviews with randomly selected experts [6–7,23–24]. The spatial data layers were classified into five criteria: environment, service functions, cultural, security, and socioeconomic. Criteria and factors were structured in a hierarchical tree and evaluated using MCDA in conjunction with GIS spatial analysis tools. Geographical phenomena often determine environmental elements, whereas cultural, security, service functions, and socioeconomic factors are influenced by human activities. The attributes of each aspect and their relevance to QoL and the nature of the raw data is illustrated in **S1 Table**. The data were collected from both primary and secondary sources. The Global Positioning System (GPS) was utilized to collect location data of the schools, healthcare facilities, postal services, security, historical sites, libraries, and religious places.

The Land Surface Temperature (LST) spatial data layer was generated by utilizing USGS Landsat8 OLI/TIRS data acquired from the USGS website (https://earthexplorer.usgs.gov/).The risk index of HEC was determined by utilizing both secondary and primary data collected in previous research conducted in the same study area. The statistical data obtained from the DS office was utilized to create density maps for power, telephone, income, sanitary facilities, and drinking water.

### Methods

The spatial indexing of QoL was conducted using a methodical flow consisting of four steps: selecting criteria, establishing a decision hierarchy, weights assignment, and deriving the QoL index.

#### Selecting criteria

The initial analysis stage involved the careful selection of criteria and factors. The study selected five categories of criteria, namely environmental, security, service functions, cultural, and socioeconomic, after assessing the previous literature. Subsequently, a total of 20 factors were integrated in order to analyse the spatial disparities in QoL and establish a spatial index for QoL within the study area.

#### Constructing the decision hierarchy

**Fig 2:**
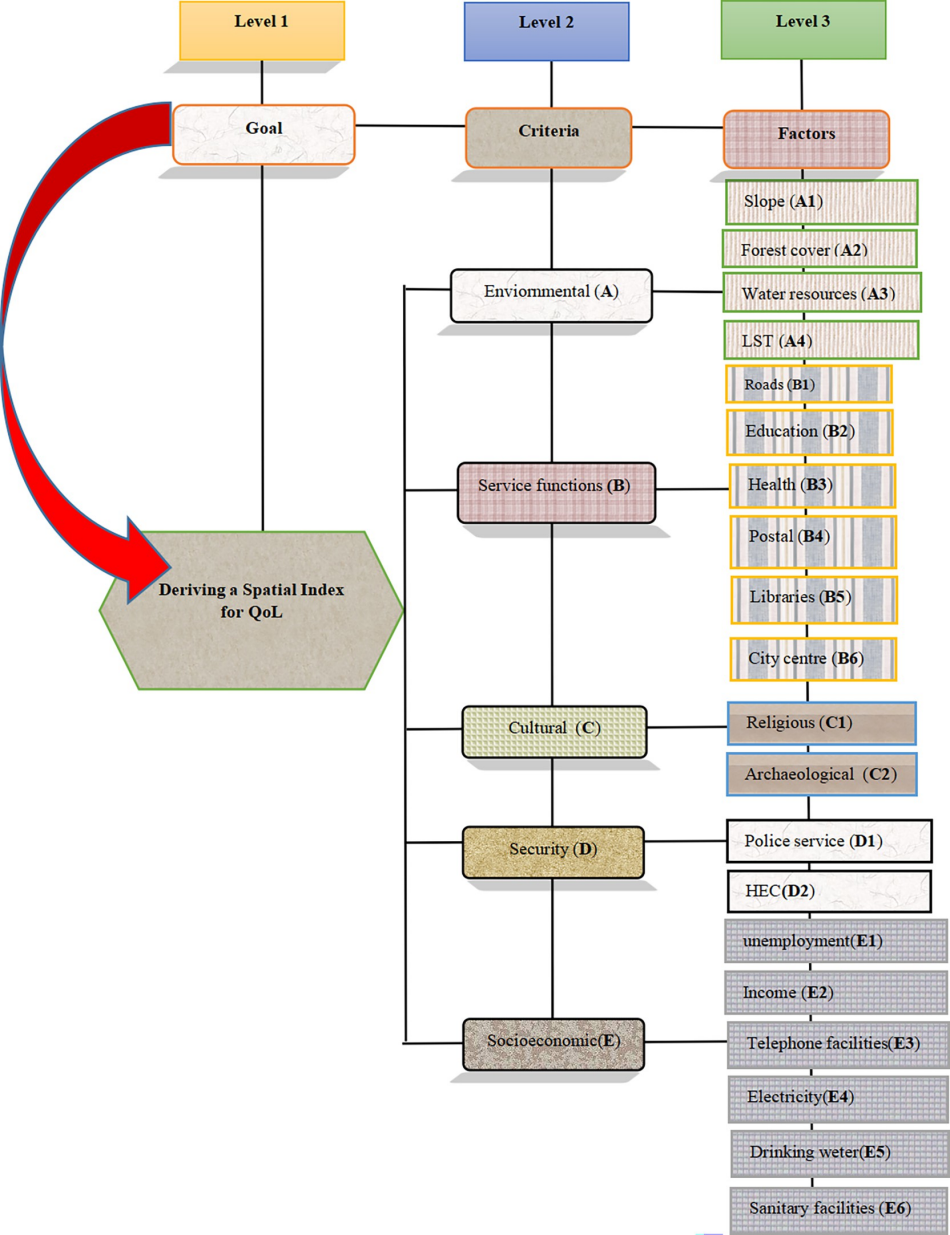
AHP structure of QoL analysis in Polpitigama DS.

To conduct the GIS-MCDA, it is important to organize and integrate the goal, criteria, and factors in a hierarchical framework. Following a thorough review of relevant literature and an examination of the background of the study area, the next phase was constructing an AHP framework. This framework was utilized to facilitate the analysis of spatial data and ultimately provide a QoL index for the area. The criteria and components were organized into a three-level hierarchical structure (**Fig 2**) during the process of creating the QoL index. The research aimed to achieve the goal at the first level of the decision hierarchy, while the second level consisted of five criteria. The third level consists of 20 factors that have been taken into account for the evaluation of QoL.

Weight assignments for criterion and factors should be conducted in a methodical manner. Pair-wise comparison matrices were utilized to ascertain the significance of each criterion and factor. The weights were derived from the expert opinion poll conducted using a semi-structured questionnaire. The experts were chosen based on their expertise in specific professional fields, particularly their research interests. Consequently, a panel of 10 experts specializing in GIS, sociology, geography, public policy, and planning provided their input on the prioritization and ranking of criteria and factors.

#### Weights assignment for criteria and factors

The experts were asked to prioritize the criteria and factors for evaluating and categorizing the QoL in the study area using Satty’s 1–9 ratio scale. The judgement values of 10 experts were combined into a group judgment using the geometric mean, which is in line with the judgment and priority [14,26]. Following the process of normalizing each value in the pairwise comparison matrix by dividing it by the sum of the column values, weights were determined using the arithmetic mean approach [30]. The geometric mean values were subsequently utilized to construct the pairwise comparison matrix. Calculating the Consistency Ratio (CR) is essential in AHP-based studies to assess the degree of discrepancy in experts’ judgment. This is done after finding the weights for each criterion. Utilizing the identical index established by Satty [14], the consistency was computed using the Eq (1);

CR=CI÷RI×100 (1)

In the equation *CR* is the consistency ratio, *CI* is the ratio of consistency index and *RI* is the random inconsistency index. In the equation of consistency ratio (*CR*), *CI* was derived using the Eq (2);

CI=(λ−n)÷(n−1) (2)

If the *CR* numeral is less than 10% it can be acceptable and if it is higher than 10%,it means that the opinion of the expert is inconsistent. The derived CR value was lower than 10% and which means that the criterion weights are reasonable and acceptable.

The AHP method computed the consistency index and ratio for the 5×5 matrix of the main criteria to be 0.0487 and 0.0434, respectively. The consistency ratio was 0.0561, whereas the consistency index for the 5×5 matrix of socioeconomic factors was 0.0695. The 0.0985 consistency index and 0.0794 consistency ratio were reported for 5×5 service factors matrix. The consistency index and consistency ratio for a 4×4 matrix of environmental factors were 0.0654 and 0.0726, respectively. 1×1 security and cultural service matrices both returned consistency scores of negative infinity and zero.

#### Deriving the QoL index

The spatial analysis extension capabilities of Arc Map10.8 streamline modelling calculations provide a convenient environment for displaying different criteria and factors as raster and vector data sets. The purpose of the study was achieved by following a series of steps. The establishment of a spatial database marked the first phase. Subsequently, the weights and rating scores generated by the AHP and the findings of the questionnaire survey were utilized to reclassify all spatial data layers pertaining to the QoL. To calculate the QoL, the index weights that obtained were multiplied by their respective variables, and all the elements were then aggregated into a single layer. The QoL index was calculated using the Eq (3) [23];

$$QoLI=\sum_{i=1}^{n=20}x_{i}w_{i}$$ (3)

Here *QoLI* is the quality of life index, *xi* is factor *i*, and *wi* is the weight of factor *i*.

The QoL index is determined by dividing the area into four zones based on specific threshold values for factors as shown in **S2 Table**. These zones range from high QoL (HQoL) to the least QoL (LEQoL). The location that most effectively fulfil all requirements is the one with the highest quality of life. Upon meeting each requirement, the other two intermediate zones were also classified as Moderate Quality of Life (MQoL) and Low Quality of Life (LQoL).

## Results

### Criteria weight and consistency

The AHP analysis indicated that the socioeconomic criteria had a greater weight, specifically 0.3721 (**Table 1**). The pairwise comparison matrix for the main criteria and factors is presented in **S3–S8 Tables**, while the normalization of the main criteria and factors is provided in **S9–S14 Tables**. In the study area, service function rated as the third most important criterion, with a weight of 0.2182. On the other hand, security criterion dropped to second place, with a weight of 0.2673. Environmental and cultural factors were assigned minimal importance in the AHP computation.

**Table 1:** Weight assignment to criteria for QoL indexing.

Criteria	weight	Factor	weight
Environmental	0.0930	Slope	0.2725
		Forest cover	0.1274
		Water resource	0.5333
		LST	0.0666
Service function	0.2182	Roads	0.1414
		Education	0.3341
		Health	0.3341
		Postal	0.0479
		Libraries	0.0497
		City centre	0.0925
Cultural	0.0490	Religious	0.7509
		Archaeological	0.2490
Security	0.2673	Police service	0.1666
		HEC	0.8333
Socio economic	0.3721	unemployment	0.1829
		Income	0.2397
		Telephone	0.0481
		Electricity	0.0833
		Drinking water	0.3075
		Sanitary facilities	0.1383

The socioeconomic criteria assigned the maximum weight (0.3075) to the availability of clean drinking water, while the percentage of fixed telephone usage received a lower weight (0.0481). The security criteria were computed by assigning a high weight of 0.8333 to the probability of human-elephant conflicts, while a weight of 0.1666 was allocated to the proximity to police stations. The religious places in closest proximity obtained the highest weight (0.7509), whereas cultural facilities, specifically archaeological sites, were assigned a weight of 0.2490 during the evaluation process. The service functions assigned the highest weight, 0.3341, to both schools and healthcare institutions. Libraries and post offices, on the other hand, were given weights of 0.0479 and 0.0497, respectively. The environmental criteria assigned the highest weight (0.5333) to the proximity to surface water resources, while the lowest weight (0.0666) was given to the LST.

### Spatial variations of QoL factors

**Fig 3:**
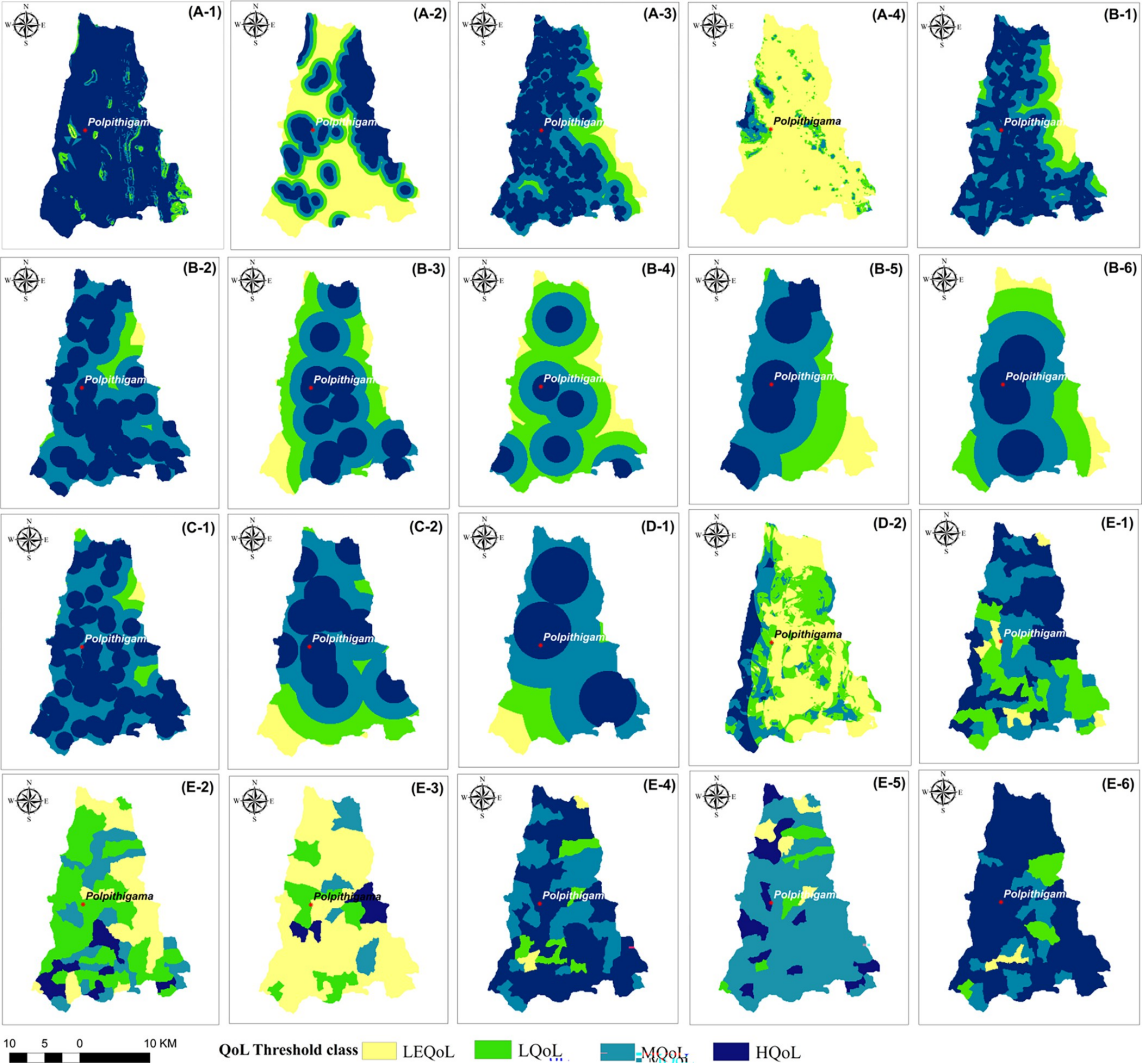
QoL threshold class maps: **(A-1)** Slope; **(A-2)** Proximity to forest; **(A-3)** Proximity to tanks; **(A-4)** LST; **(B-1)** Proximity to roads; **(B-2)** Proximity to schools; **(B-3)** Proximity to hospitals; **(B-4)** Proximity to postal facilities; **(B-5)** Proximity to library;**(B-6)** Proximity to growth centers; **(C-1)** Proximity to religious places; **(C-2)** Proximity to archaeological sites; **(D-1)** Proximity to police stations; **(D-2)** Human Elephant conflict risk; **(E-1)** Unemployment %; **(E-2)** Household income; **(E-3)** Fixed telephone facilities; **(E-4)** Electricity facilities; **(E-5)** Drinking wells; **(E-6)** Sanitary facilities. Maps were edited by authors using United States Geological Survey Earth Explorer Landsat images [32].

The spatial variations of QoL in the DS for each of the 20 factors were illustrated in **Fig 3**. Due to the flat topography of the study area, with heights ranging from 77m to 506m above mean sea level, except for the southeastern portion which features mountains, the QoL is greater. Eastern border is home to the majority of its natural forests and protected areas. Consequently, these localities exhibit a higher standard of living compared to the western half.

Therefore, when near dense natural forests, most locations in the western part are classified as LEQoL. Several tanks are distributed throughout the western region of the study area, serving as surface water resources for agricultural and domestic purposes. This portion has a greater QoL index compared to the eastern section, which has a least and low QoL. The study area is often located in the dry part of the Kurunegala district, adjacent to the Anuradhapura district. Consequently, the QoL is typically rated as being in the low and least categories, based on the LST. The presence of forest areas has resulted in the LEQoL classes in areas close to roads. However, the dense road network in the western and southern parts of DS has significantly contributed to high QoL. These regions cover 61% (245 km^2^) of the total area and just 11% (44 km^2^) falls within the least QoL class. As a result of the limited number of government schools in the eastern region, the QoL in terms of school proximity is generally rated as very poor, covering an area of 164 km^2^ (41%). In contrast, only 36 km^2^ (9%) in the southern and central portions of the DS are classified as having HQoL in this regard.

Exception of Polpitigama Town, the health facilities classify Galtanwewa in the centre and Rambe in the south as areas with low and the least QoL respectively. The majority of the study area is devoid of postal services, with the exception of Polpitigama Town, Madagalla, Galtanwewa, and Rambe Junction. Approximately 70% of regions fall into the LQoL category due to inadequate library facilities. Almost 75% of localities fall into the high QoL category based on their closeness to cultural amenities. The majority of the DS is within the LEQoL category upon the proximity to police stations.

The eastern side of DS poses the highest danger of Human-Elephant Conflict due to its connection to the Kahalla-Pallekele (KPK) elephant corridor. Therefore, the areas with LQoL and the LEQoL encompassed a bigger portion of 221 km^2^ (56%). Considering the HEC risk, only small areas in the western and southwestern parts fall inside the HQoL zone, which spans an area of 21km^2^. The majority of GNDs have the HQoL when socioeconomic variables are taken into account. Nonetheless, most of the areas are in the zone with the LEQoL in terms of monthly income and fixed telephone connectivity. For instance, upon fixed telephone connection facility the LEQoL zone contributed 70% (282 km^2^) while the HQoL zone accounted for 6% (25 km^2^). Given the significant proportion of households with a monthly income of less than Rs.10000, the majority of the GNDs fall into the least and low QoL category, with the exception of four GNDs located in the southern part.

### Quality of life index for Polpitigama DS

**Fig 4:**
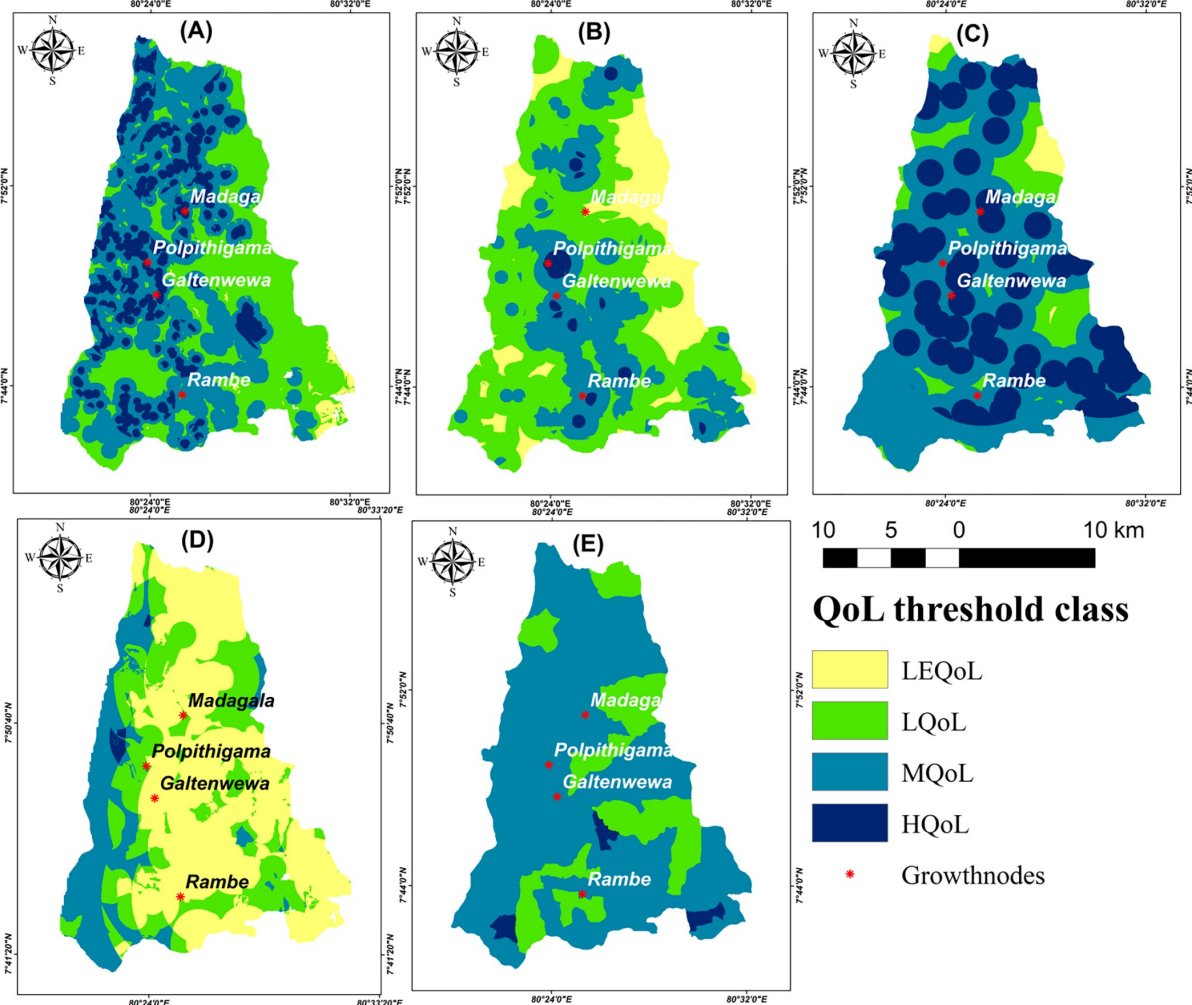
Spatial variations maps of QoL: Environment **(A)**; service functions **(B)**; cultural facilities **(C)**; Security **(D)**; socioeconomic factors **(E)**. Maps were edited by authors using United States Geological Survey Earth Explorer Landsat images [32].

The QoL index was derived in the DS by integrating AHP and MCDA, through the overlay of five criteria. **Fig 4** revealed varying levels of QoL for different criteria ranging from high to low.

In addition, Arc Map 10.8 software calculated descriptive data for the area coverage in both km^2^ and the percentage contribution of each QoL class as **Table 2**. The cultural facility, which covered an area of 148 km^2^, accounted for 36.9% of the HQoL zone. It had the greatest percentage among all the criteria in this class. The areas with the LEQoL in terms of cultural facilities are limited to small patches in the northeast and northern areas. The minimum area coverage for the HQoL class was determined to be 0.7% (2.5 km^2^) based on security factors. Most communities, excluding the western section, have a LEQoL mostly because of the presence of the DS in the HEC risk area. The security criterion accounted for the biggest percentage (54.7%) of the LEQoL among the five criteria, covering an area of 216 km^2^. On the other hand, the environment criteria had the lowest contribution (1.4%) to the LEQoL. Furthermore, there are no areas that fall into the least quality of life category based on socioeconomic criteria, and most of the GNDs are situated in the moderate quality of life zone, accounting for 76.6%.

**Table 2:** Descriptive statistics of the QoL index.

**a.** Area (km ^2^) by criteria						
**QoL class**	Overall QoL	EnviQoL	Service QoL	Cultural QoL	Security QoL	SocEco QoL
LEQoL	54.9	5.3	68.6	7.9	216	none
LQoL	252	132	216	42.9	107.9	85
MQoL	70	186.3	107.9	201.9	68.4	301.6
HQoL	17.3	70.5	8.5	148.3	2.5	8.5
**Total**	394.2	394.1	394.1	394.1	394.8	394.1
**b.**Area (%) by criteria						
**QoL class**	Overall QoL	Envi QoL	Service QoL	Cultural QoL	Security QoL	SocEco QoL
LEQoL	13.9	1.4	17.1	1.9	54.7	none
LQoL	63.8	33.6	53.8	10.7	27.3	21.2
MQoL	17.8	47.2	26.9	50.5	17.3	76.6
HQoL	4.5	17.8	2.2	36.9	0.7	2.2
**Total**	100	100	100	100	100	100

**Fig 5:**
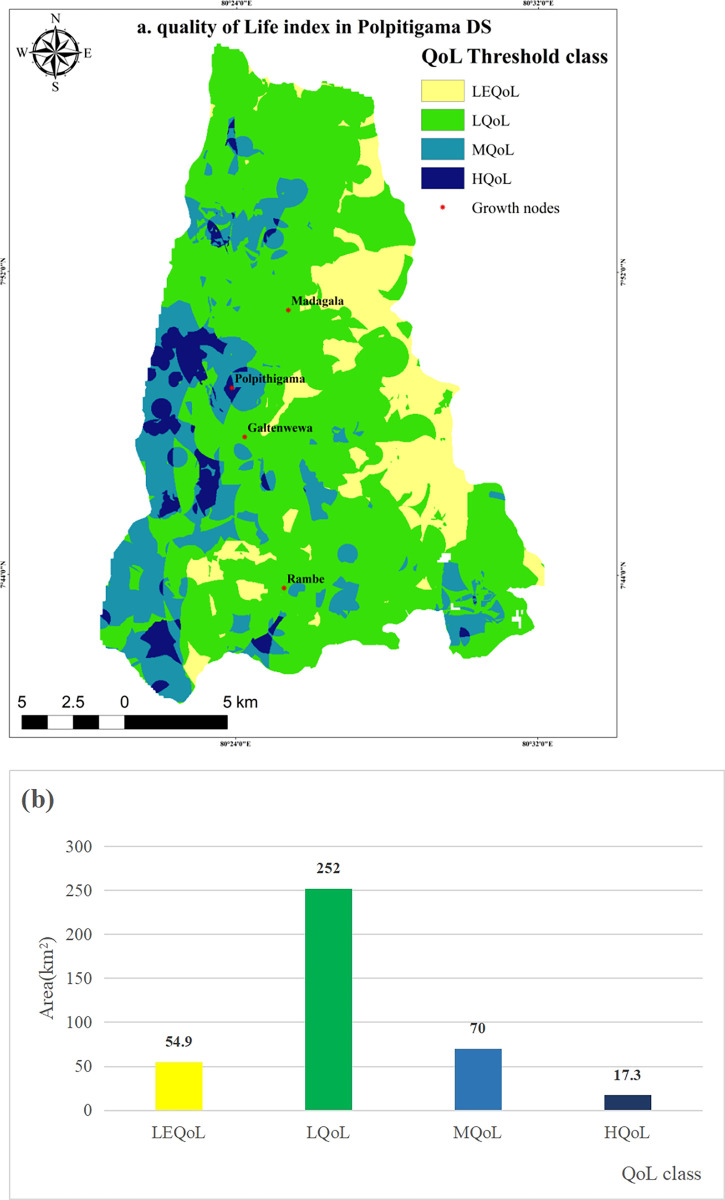
Quality of Life index: **(a)** QoL index map in Polptigama DS; **(b)** Descriptive statistics of QoL index. Map was edited by authors using United States Geological Survey Earth Explorer Landsat images [32].

The areas of Polpitigama, Madagalla, and Galtanwewa have become highly desirable locations due to the abundance of service facilities, resulting in a good quality of life. However, by amalgamating each criterion, these separate conclusions were distinguished. The results revealed that a mere 4.5% (17.3 km^2^) of the total area of 394 km^2^ met the criteria for high quality of life. In contrast, the majority of the area, accounting for 63.8% (252 km^2^), fell into the low quality of life category (**Fig 5**). The zone with moderate quality of life accounted for 17.8%, while the zone with the least quality of life accounted for 13.9%.

## Discussion

### Effect of town distance on QoL

**Fig 6:**
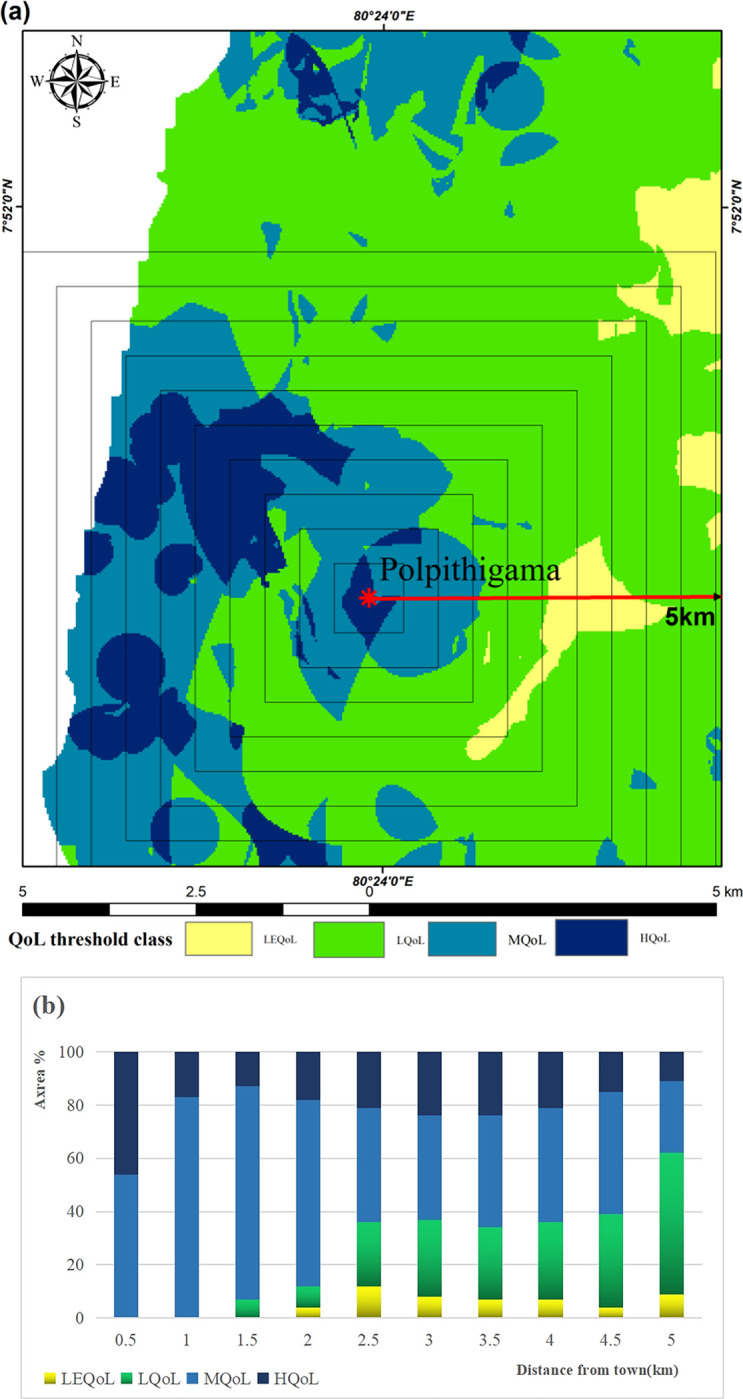
Distance effect for QoL: **(a)** Variations of Quality of Life (QoL) from the town center to the periphery; **(b)** Percentage contribution of QoL gradient zones. Map was edited by authors using United States Geological Survey Earth Explorer Landsat images [32].

The agglomeration effect leads to the concentration of service functions and infrastructure facilities in towns and cities. Hence, a distance-based gradient analysis was conducted to investigate whether the town centre had any influence on the QoL. In order to examine the influence of distance from Polpitigama Town on spatial variations in QoL, ten gradient zones were created with intervals of 0.5 km (**Fig 6A**). The 5 km gradient zone has an area of 90 km^2^. Based on the coverage of each QoL class, the MQoL zone covers 48.5 km^2^, the HQoL zone covers 10.5 km^2^, and the low and least QoL zones cover 28.3 km^2^ and 2.9 km^2^ correspondingly.

The gradient analysis indicates that the high quality of life zone’s influence is decreasing as one moves out from the center of the town (**Fig 6b**). The zone with a least quality of life is progressively growing in size. The areas with the lowest quality of life within the study region have fluctuations, but generally show an upward trend. Approximately 70% of the areas with moderate and high quality of life were located within a 2 km radius from the town center. However, this number declined by approximately 40% while moving 5 km away from the town.

### Validating the QoL index

**Fig 7:**
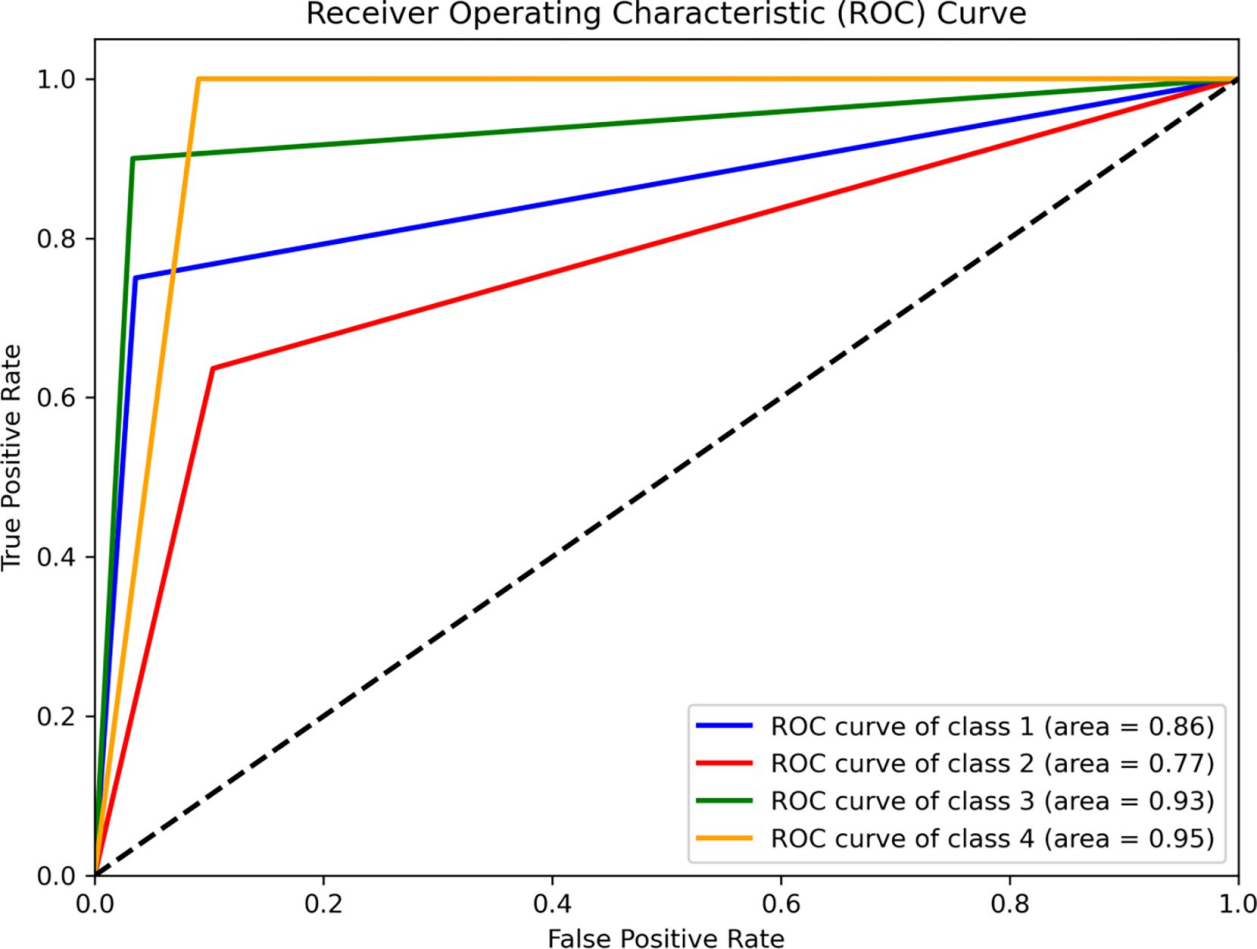
Receiver operating characteristic (ROC) curve of the model validation.

Validating the QoL index was essential for the reliability of the derived results [33–35]. The receiver operating characteristic (ROC) curve is the most generally used strategy that illustrates the correlation between false positive values (Y-axis) and false negative values (X-axis) as used in the investigation also (**Fig 7**) [33–37].

The prediction accuracy of the ROC curve is demonstrated by the area under the curve (AUC) that describes the absence and presence of the events [33–35,38]. For validation 40 random points were collected from the current QoL zones and compared with the generated QoL map (**S2 Fig**). Out of 40 sample points 32 locations perfectly fit with the obtained QoL classes (**S15 Table**). The ROC curve obtained shows high AUC values: 0.95 for LEQoL areas, 0.93 for MQoL, 0.86 for HQoL, and 0.77 for LQoL. This indicates an overall prediction accuracy of 87.7%. In summary, resulted AUC value suggests that the AHP-based QoL analysis is better than random chance. In the absence of prior research the resulted index is credible and may be applied as a road map for the livelihood improvement of poor households in the study area.

### Equitable resource allocation to improve QoL

The findings indicate that the majority of the areas in Polpithigama are characterized by low and moderate QoL. Socioeconomic factors exert a greater influence than environmental, cultural, and safety factors. The results align with the findings made by Dissanayake et al. [23] in the city of Kandy, Sri Lanka. The data indicated that socioeconomic factors had a significant impact on the quality of life in the majority of the region. Therefore, policymakers should prioritize the provision of employment opportunities, electricity, drinking water, housing, and sanitary facilities for the marginalized people, particularly those residing in rural areas. However, whereas transportation was identified as a significant component in the study conducted by Dissanayake et al. [23], the current data indicate that schools and healthcare facilities have a greater impact on QoL. The study conducted in the municipality of Katerini, Greece [8] also discovered that service factors play a vital role in determining the QoL. The town significantly influences the study area’s quality of life. The proximity of the town is a significant factor affecting the levels of life quality. This aligns with the findings of Dissanayake et al. [23], which indicate that as the distance between settlements and the town increases to approximately 5km, the life quality also decreases. Faka et al., [24], found comparable results in Athens city, suggesting that the urban center provides more advantages than the rural outskirts due to the strategic location of service facilities. Rural residents face hindered prosperity as a result of inadequate provision of essential amenities and services, including easily accessible schools, healthcare facilities and retail establishments. In many impoverished rural areas, the absence of adequate housing, transportation, and access to safe and sufficient drinking water poses significant challenges in meeting basic needs. Planners and rural societies can employ adaptable strategies to engage economically disadvantaged and fatigued individuals who face barriers to participation, are typically not involved in government procedures, or are disproportionately affected by development initiatives. Given that the HEC risk is the most significant factor in determining safety standards, decision-makers should prioritize minimizing HEC risk and developing more effective methods to safeguard both elephants and humans.

### Limitations and future research direction

The research only undertook 20 criteria into account based on earlier scholars [8,23,24]. Other factors were not included in this due to time and data limitations.Therefore, the study was limited to a small area due to limitations in time and resources. Identifying the QoL is a crucial matter due to its significant influence on underdeveloped regions within the district. Therefore, it is necessary to conduct more extensive research in the future to explore the quality of life in remote areas with limited socioeconomic and service infrastructure by consolidating intricate criteria and factors. Conducting comparative studies with other established MCDA methodologies, such as TOPSIS and DELPHI, will yield more realistic results in the future, in addition to model validation.

## Conclusions

This research was conducted to derive a QoL index for a Divisional Secretariat located in a rural periphery of Sri Lanka using GIS integrated MCDA techniques. Spatial variables allows for the mapping of factors and criteria, facilitating the identification of spatial patterns associated with low or high QoL. Socioeconomic and service factors were shown to have greater significance than environmental and cultural variables. Based on the gradient zone analysis, the area surrounding the town of Polpitigama exhibits the highest QoL. Furthermore, the distance from the town has a notable influence on the quality of life. The results indicate that improving socio-economic infrastructure and service functions, such as hospitals and schools, can enhance the quality of life in rural areas. Model validation was more useful in maintaining the scientific reliability of the resulting index through ROC curve and it has shown a good consistency of the derived model with real world scenario. Furthermore, policymakers should prioritize the implementation of initiatives to minimize the adverse effects of HEC in their local land use planning. Although, not all criteria are applicable, the same approach remains adaptable. Consequently, this approach can be utilized to assess the QoL in most rural areas in Sri Lanka.

## Supporting information

S1 Fig Common factors/aspects of QoL. (TIF)

S2 Fig Field verification samples used for model validation. (TIF)

S1 Table Factors used for the spatial analysis of QOL and their relevance, type, and sources. (DOCX)

S2 Table Factor threshold and QoL classes. (DOCX)

S3 Table Pairwise comparison matrix for main criteria. (DOCX)

S4 Table Pairwise comparison matrix for environment factors. (DOCX)

S5 Table Pairwise comparison matrix for service facilities. (DOCX)

S6 Table Pairwise comparison matrix for cultural facilities. (DOCX)

S7 Table Pairwise comparison matrix for security facilities. (DOCX)

S8 Table Pairwise comparison matrix for socioeconomic facilities. (DOCX)

S9 Table Main criteria normalization. (DOCX)

S10 Table Environment factors normalization. (DOCX)

S11 Table Service factors normalization. (DOCX)

S12 Table Cultural factors normalization. (DOCX)

S13 Table Security factors normalization. (DOCX)

S14 Table Socioeconomic factors normalization. (DOCX)

S15 Table Information of field data and pixel correlation with suitability map. (DOCX)
