# Supplementary figures and images for: A quality of life index for the rural periphery of Sri Lanka using GIS multi-criteria decision analysis techniques
Source: PLoS One. 2024 Sep 18;19(9):e0308077. doi: 10.1371/journal.pone.0308077 (PMC11410255; doi:10.1371/journal.pone.0308077)

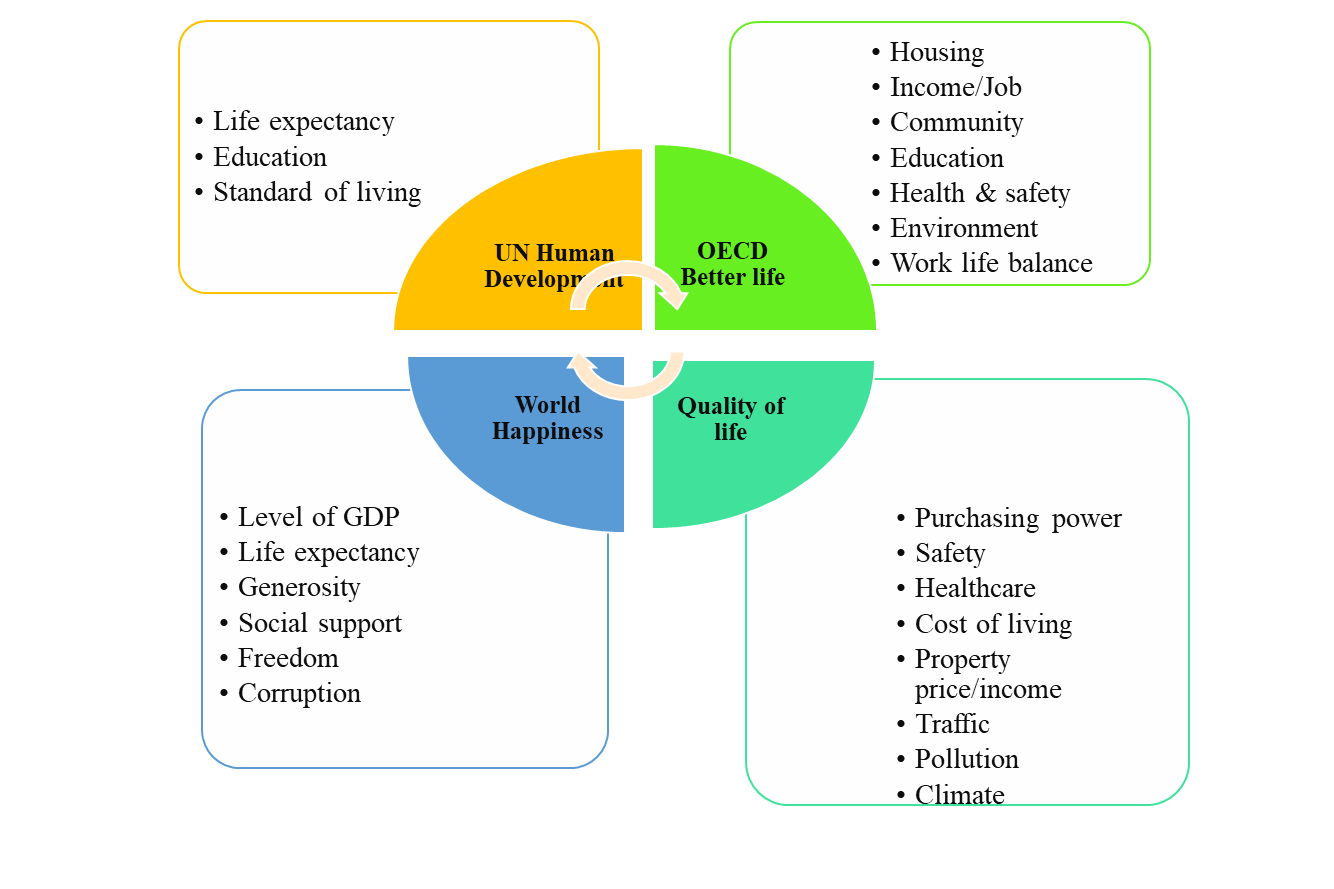

Supplement: S1 Fig — (TIF) [file pone.0308077.s001.tif]

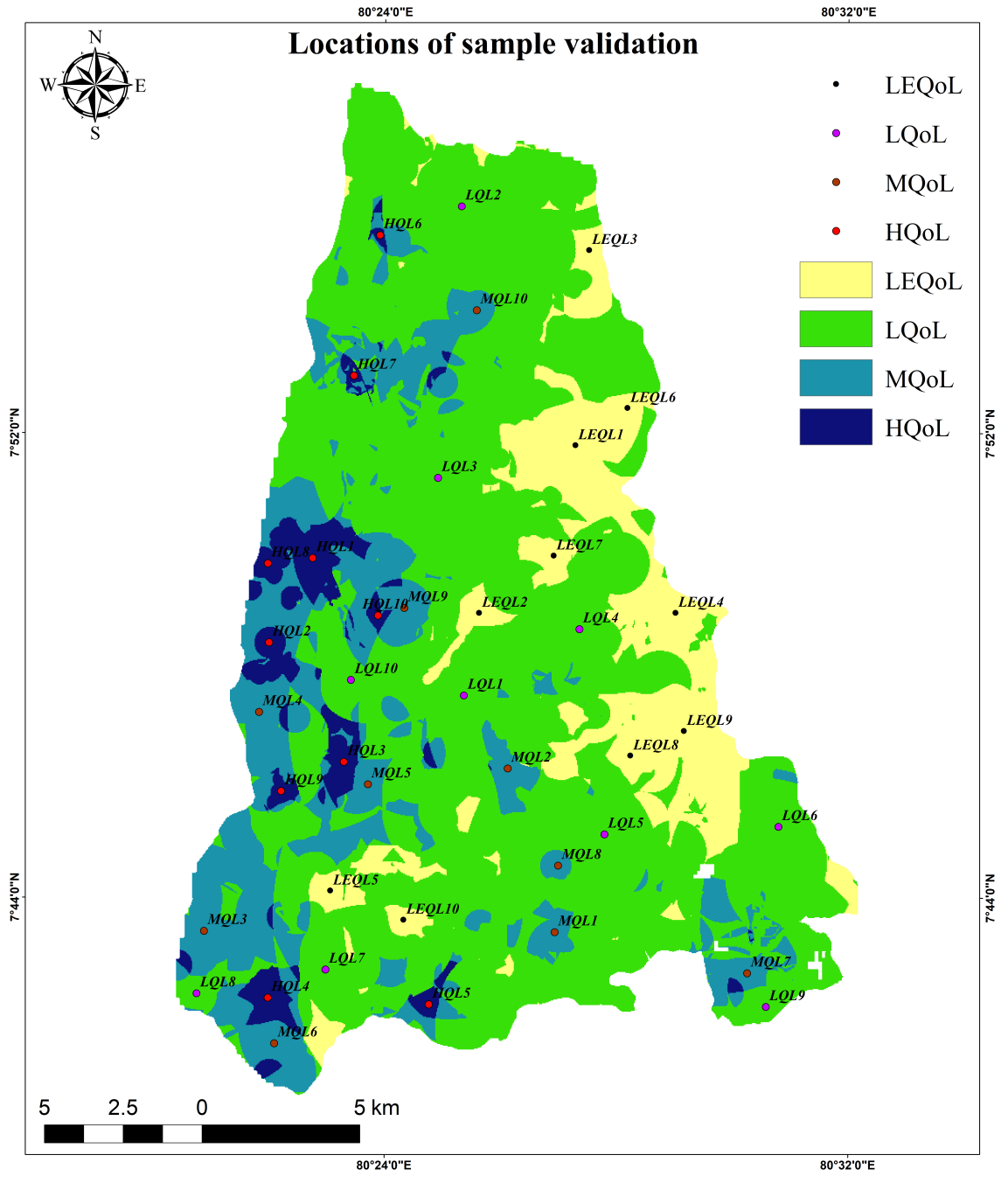

Supplement: S2 Fig — (TIF) [file pone.0308077.s002.tif]
