# Supplementary material for: A quality of life index for the rural periphery of Sri Lanka using GIS multi-criteria decision analysis techniques
Source: PLoS One. 2024 Sep 18;19(9):e0308077. doi: 10.1371/journal.pone.0308077 (PMC11410255; doi:10.1371/journal.pone.0308077)
Supplement: S1 Table — (DOCX) [file pone.0308077.s003.docx]

| **Factor** | **Relevance for QoL** | **Raw Data** | **Data processing** |
| --- | --- | --- | --- |
| Urban center* | For residents in neighboring regions, urban centers offer additional infrastructure, service functions, and livelihood alternatives. As the distance from urban centers increases, the availability of services will diminish, which could have a bad effect on QOL. | Point data in vector format | Process vector shape file using Arc Map proximity analysis tool |
| Education * | A person with a good education has more chances to improve his life and maintain good health. Through civic engagement that works to reduce poverty and crimes in a certain location, more advantages can be distributed to society (Onnom et al.2018). | Government schools Point data in vector format | Process vector and raster files using the Arc Map proximity analysis tool |
| Health * | Good health of an individual secures a long life that is free from chronic and long-term diseases. Health is important to self-esteem and self-image as well (Onnom et al.2018). | Government and private hospitals and medical centers Point data in vector format |  |
| Postal service* | The Postal Service, which facilitates communication between individuals and promotes informed citizenship. And its contribution to coordinating government services and institutes. | Post and sub-post office Point data in vector format |  |
| Transportation** | Road networks are essential for the mobility of people and resources. The service provision of a region is directly affected by transportation. Settlement location also depends on accessibility (Dissanayake et al.2020; Sze and Christensen, 2017). | Major and minor roads line data in raster format |  |
| Libraries* | Play an important role in supporting education and literacy. They provide countless resources by disseminating knowledge and information to individuals through educational materials, scientific publications, and newspapers. | Location of Libraries Point data in vector format |  |
| Religious places* | Participating in spiritual and religious activities will aid a person in achieving better health outcomes, such as increased longevity, coping mechanisms, and health-related quality of life (Behere et al.2013; Dissanayake et al.2020). | Locations of temples, churches, and mosques Point data in vector format |  |
| Archaeology and heritage* | The historical locations and sites might help the scenery create a pleasing atmosphere. Additionally, they increased the value of the physical surroundings (Dissanayake et al.2020). | Locations of historical sites, monuments Point data in vector format | Process vector and raster files using Arc Map proximity analysis tool |
| Forest cover** | High-density forest cover improves the environment's aesthetic value and scenic appeal, and it also has a positive impact on an individual's spiritual health and ensures pleasant living conditions (Dissanayake et al.2020; Maas et al.2009). | Natural Forest and protected areas data in raster format |  |
| Land Surface Temperature (LST) ** | Extreme temperatures affect QOL. A greater incidence of sickness, especially deteriorated chronic illnesses like cardiovascular, pulmonary, and cerebrovascular disease, is linked to variations in the average temperature. | Landsat-8OLI/TIRS raster data | Preparation of different LST surfaces |
| Surface water resources* | Humans rely on water for food, agriculture and livestock | Tanks, ponds, reservoirs Polygon data in vector format | Process vector shape files using Arc Map proximity analysis tool |
| Slope* | The places with high slopes and elevations are not suited for habitation. Settlements built on steep slopes are prone to landslides and have expensive construction costs. | DEM | Slope map using elevation points and DEM |
| Security* | Situational awareness is cultivated by security. Without security, people frequently feel complacent and fail to notice odd conduct by nearby people | Police station and police post Point data in vector format | Process vector shape files using Arc Map proximity analysis tool |
| Human Elephant Conflicts (HEC)* | As a result of HEC individuals may suffer death or physical injury, or property loss, including homes and croplands. Such tensions put a person at danger for their mental well-being as well. | HEC risk areas | Preparation of HEC risk index using Arc Map spatial analysis. |
| Electricity facilities*** | In order to create healthy interior environments with healthy air temperatures, humidity levels, noise levels, and enhanced air quality, humans need access to abundant, affordable, secure, and safe energy(https://www.iea.org). | Density maps | Preparing density layers for each criterion on the % of individual/household unit |
| Sanitary facilities*** | Due to negative effects including anxiety, a higher chance of sexual assault, and missed opportunities for employment and education, poor sanitation hinders social and economic development as well as human well-being (https://www.who.int). |  |  |
| Income *** | It directly affects health through the products and services that people purchase, which can either improve or deteriorate their health (http://www.healthscotland.scot). |  |  |
| Drinking water*** | Several diseases, including cholera, diarrhoea, dysentery, hepatitis A, typhoid, and polio, are associated with the transmission of contaminated water. Water that is absent, insufficient, or improperly managed puts people at risk for health problems that may be avoided (https://www.who.int). |  |  |
| Telephone facilities*** | Communication is essential for community interaction and the diffusion of innovation over the space. |  |  |
| Employment*** | A good-paying job makes it easier for humans to live in healthier and long life as well as more access to service and mental health (https://www.rwjf.org) |  |  |
