# Supplementary material for: A quality of life index for the rural periphery of Sri Lanka using GIS multi-criteria decision analysis techniques
Source: PLoS One. 2024 Sep 18;19(9):e0308077. doi: 10.1371/journal.pone.0308077 (PMC11410255; doi:10.1371/journal.pone.0308077)
Supplement: S2 Table — (DOCX) [file pone.0308077.s004.docx]

| **QoL Factor** | **Threshold classes** | | | |
| --- | --- | --- | --- | --- |
|  | **LEQoL** | **LQoL** | **MQoL** | **HQoL** |
| **Environmental(A)** | | | | |
| Slope **(A1)** | 30^0^> | 11-30^0^ | 5-11^0^ | <5^0^ |
| Distance to Forest **(A2)** | >1.5km | 1-1.5km | 0.5-1km | <0.5km |
| Distance to Water resources **(A3)** | >0.75km | 0.5-0.75km | 0.25-0.5km | <0.25 km |
| LST **(A4)** | >60^0^C | 55-60^0^C | 50-55^0^C | <50^0^C |
| **Service functions(B)** | | | | |
| Distance to Roads **(B1)** | >1.5km | 1-1.5 km | 0.5-1km | <0.5km |
| Distance to schools **(B2)** | >1.5km | 1-1.5km | 0.5-1km | <0.5km |
| Distance to health facilities **(B3)** | >3km | 2-3km | 1-2km | <1km |
| Distance to post office **(B4)** | >6km | 4-6km | 2-4km | <2km |
| Distance to libraries **(B5)** | >3km | 2-3km | 1-2km | <1km |
| Distance to City **(B6)** | >10km | 5-10km | 2.5-5km | <2.5km |
| **Cultural (C )** | | | | |
| Distance to Religious places **(C1)** | >3km | 2-3km | 1-2km | <1km |
| Distance to archaeological sites **(C2)** | >6km | 4-6km | 2-4km | <2km |
| **Security (D)** | | | | |
| Distance to police station **(D1)** | >3km | 2-3km | 1-2km | <1km |
| HEC risk zone **(D2)** | Very high risk | High risk | Moderate risk | Low/Least risk |
| **Socioeconomic (E )** | | | | |
| Unemployment **(E1)** | >24% | 16-24% | 8-16% | <8% |
| Income **(E2)** | <25% | 25-50% | 50-75% | >75% |
| Telephone facilities **(E3)** | <8% | 8-16% | 16-24% | >24% |
| Electricity **(E4)** | >6% | 4-6% | 2-4% | <2% |
| Drinking water **(E5)** | <10% | 10-25% | 25-75% | >75% |
| Sanitary facilities **(E6)** | >12% | 8-12% | 4-8% | <4% |
