# Supplementary material for: A quality of life index for the rural periphery of Sri Lanka using GIS multi-criteria decision analysis techniques
Source: PLoS One. 2024 Sep 18;19(9):e0308077. doi: 10.1371/journal.pone.0308077 (PMC11410255; doi:10.1371/journal.pone.0308077)
Supplement: S4 Table — (DOCX) [file pone.0308077.s006.docx]

|  | Slope | Forest distance | Water Resources | LST |
| --- | --- | --- | --- | --- |
| Slope | 1.00 | 0.33 | 3.00 | 0.20 |
| Forest distance | 3.00 | 1.00 | 0.20 | 0.20 |
| Water resource | 0.33 | 5.00 | 1.00 | 0.20 |
| LST | 5.00 | 5.00 | 5.00 | 1.00 |
