# Supplementary material for: A quality of life index for the rural periphery of Sri Lanka using GIS multi-criteria decision analysis techniques
Source: PLoS One. 2024 Sep 18;19(9):e0308077. doi: 10.1371/journal.pone.0308077 (PMC11410255; doi:10.1371/journal.pone.0308077)
Supplement: S5 Table — (DOCX) [file pone.0308077.s007.docx]

|  | Proximity to roads | Proximity to schools | Proximity to hospitals | Proximity to post office | Proximity to library | Proximity to towns |
| --- | --- | --- | --- | --- | --- | --- |
| Proximity roads | 1.00 | 0.20 | 0.20 | 5.00 | 3.00 | 3.00 |
| Proximity to schools | 5.00 | 1.00 | 1.00 | 5.00 | 0.20 | 0.20 |
| Proximity to hospitals | 5.00 | 1.00 | 1.00 | 5.00 | 3.00 | 5.00 |
| Proximity to post office | 0.20 | 0.20 | 0.20 | 1.00 | 1.00 | 0.33 |
| Proximity to library | 0.33 | 5.00 | 0.33 | 1.00 | 1.00 | 0.33 |
| Proximity to towns | 0.33 | 5.00 | 0.20 | 3.00 | 3.00 | 1.00 |
