# Supplementary material for: A quality of life index for the rural periphery of Sri Lanka using GIS multi-criteria decision analysis techniques
Source: PLoS One. 2024 Sep 18;19(9):e0308077. doi: 10.1371/journal.pone.0308077 (PMC11410255; doi:10.1371/journal.pone.0308077)
Supplement: S8 Table — (DOCX) [file pone.0308077.s010.docx]

|  | Employment | Monthly income | Telephone facilities | Electricity facilities | Drinking Water | Sanitary facilities |
| --- | --- | --- | --- | --- | --- | --- |
| Employment | 1.00 | 1.00 | 0.20 | 3.00 | 0.33 | 1.00 |
| Monthly income | 1.00 | 1.00 | 3.00 | 0.33 | 1.00 | 0.33 |
| Telephone facilities | 5.00 | 0.33 | 1.00 | 0.33 | 0.20 | 0.33 |
| Electricity facilities | 0.33 | 3.00 | 3.00 | 1.00 | 0.33 | 0.33 |
| Drinking water | 3.00 | 1.00 | 5.00 | 3.00 | 1.00 | 1.00 |
| Sanitary facilities | 1.00 | 3.00 | 3.00 | 3.00 | 1.00 | 1.00 |
