# Supplementary material for: A quality of life index for the rural periphery of Sri Lanka using GIS multi-criteria decision analysis techniques
Source: PLoS One. 2024 Sep 18;19(9):e0308077. doi: 10.1371/journal.pone.0308077 (PMC11410255; doi:10.1371/journal.pone.0308077)
Supplement: S9 Table — (DOCX) [file pone.0308077.s011.docx]

|  | Environment | Services | cultural | Security | socioeconomic | Normalization |
| --- | --- | --- | --- | --- | --- | --- |
| Environment | 0.07 | 0.04 | 0.05 | 0.04 | 0.06 | 0.0500 |
| Services | 0.20 | 0.12 | 0.03 | 0.12 | 0.29 | 0.1362 |
| Cultural | 0.20 | 0.60 | 0.15 | 0.60 | 0.06 | 0.2985 |
| Security | 0.20 | 0.12 | 0.03 | 0.12 | 0.29 | 0.1362 |
| Socioeconomic | 0.33 | 0.12 | 0.74 | 0.12 | 0.29 | 0.3788 |
