# Supplementary material for: A quality of life index for the rural periphery of Sri Lanka using GIS multi-criteria decision analysis techniques
Source: PLoS One. 2024 Sep 18;19(9):e0308077. doi: 10.1371/journal.pone.0308077 (PMC11410255; doi:10.1371/journal.pone.0308077)
Supplement: S10 Table — (DOCX) [file pone.0308077.s012.docx]

|  | Slope | Forest distance | Water Resources | LST | Normalization |
| --- | --- | --- | --- | --- | --- |
| Slope | 0.11 | 0.03 | 0.33 | 0.13 | 0.1693 |
| Forest distance | 0.32 | 0.09 | 0.02 | 0.13 | 0.1362 |
| Water resources | 0.04 | 0.44 | 0.11 | 0.13 | 0.1855 |
| LST | 0.54 | 0.44 | 0.54 | 0.63 | 0.5324 |
