# Supplementary material for: A quality of life index for the rural periphery of Sri Lanka using GIS multi-criteria decision analysis techniques
Source: PLoS One. 2024 Sep 18;19(9):e0308077. doi: 10.1371/journal.pone.0308077 (PMC11410255; doi:10.1371/journal.pone.0308077)
Supplement: S11 Table — (DOCX) [file pone.0308077.s013.docx]

|  | Roads | Schools | Hospitals | Post office | Library | Towns | Normalization |
| --- | --- | --- | --- | --- | --- | --- | --- |
| Roads | 0.08 | 0.02 | 0.07 | 0.25 | 0.27 | 0.30 | 0.1572 |
| Schools | 0.42 | 0.08 | 0.34 | 0.25 | 0.02 | 0.02 | 0.1693 |
| Hospitals | 0.42 | 0.08 | 0.34 | 0.25 | 0.27 | 0.51 | 0.3101 |
| Post office | 0.02 | 0.02 | 0.07 | 0.05 | 0.09 | 0.03 | 0.0405 |
| Library | 0.03 | 0.40 | 0.11 | 0.05 | 0.09 | 0.03 | 0.1371 |
| Towns | 0.03 | 0.40 | 0.07 | 0.15 | 0.27 | 0.10 | 0.1855 |
