# Supplementary material for: A quality of life index for the rural periphery of Sri Lanka using GIS multi-criteria decision analysis techniques
Source: PLoS One. 2024 Sep 18;19(9):e0308077. doi: 10.1371/journal.pone.0308077 (PMC11410255; doi:10.1371/journal.pone.0308077)
Supplement: S12 Table — (DOCX) [file pone.0308077.s014.docx]

|  | Religious places | Archaeological sites | Normalization |
| --- | --- | --- | --- |
| Religious places | 0.25 | 0.25 | 0.2499 |
| Archaeological sites | 0.75 | 0.75 | 0.7499 |
