# Supplementary material for: A quality of life index for the rural periphery of Sri Lanka using GIS multi-criteria decision analysis techniques
Source: PLoS One. 2024 Sep 18;19(9):e0308077. doi: 10.1371/journal.pone.0308077 (PMC11410255; doi:10.1371/journal.pone.0308077)
Supplement: S13 Table — (DOCX) [file pone.0308077.s015.docx]

|  | Police stations | HEC risk | Normalization |
| --- | --- | --- | --- |
| Police stations | 0.17 | 0.17 | 0.1666 |
| HEC risk | 0.83 | 0.83 | 0.8333 |
