# Supplementary material for: A quality of life index for the rural periphery of Sri Lanka using GIS multi-criteria decision analysis techniques
Source: PLoS One. 2024 Sep 18;19(9):e0308077. doi: 10.1371/journal.pone.0308077 (PMC11410255; doi:10.1371/journal.pone.0308077)
Supplement: S14 Table — (DOCX) [file pone.0308077.s016.docx]

|  | Employment | Monthly income | Telephone | Electricity | Drinking water | Sanitary facilities | Normalization |
| --- | --- | --- | --- | --- | --- | --- | --- |
| Employment | 0.09 | 0.11 | 0.01 | 0.28 | 0.09 | 0.25 | 0.1276 |
| Monthly income | 0.09 | 0.11 | 0.20 | 0.03 | 0.26 | 0.08 | 0.1273 |
| Telephone | 0.44 | 0.04 | 0.07 | 0.03 | 0.05 | 0.08 | 0.1238 |
| Electricity | 0.03 | 0.32 | 0.20 | 0.09 | 0.09 | 0.08 | 0.1388 |
| Drinking water | 0.26 | 0.11 | 0.33 | 0.28 | 0.26 | 0.25 | 0.2569 |
| Sanitary facilities | 0.09 | 0.32 | 0.20 | 0.28 | 0.26 | 0.25 | 0.2255 |
