# Supplementary material for: A quality of life index for the rural periphery of Sri Lanka using GIS multi-criteria decision analysis techniques
Source: PLoS One. 2024 Sep 18;19(9):e0308077. doi: 10.1371/journal.pone.0308077 (PMC11410255; doi:10.1371/journal.pone.0308077)
Supplement: S15 Table — (DOCX) [file pone.0308077.s017.docx]

| **Field condition** | | | | | **QOL map** | |
| --- | --- | --- | --- | --- | --- | --- |
| **Site no** | Latitude | Longitude | QOL scale | Description | Suitability scale | Remark |
| LEQL1 | 7°51'47"N | 80°27'17"E | 1 | Least quality | 1 | true |
| LEQL2 | 7°48'54"N | 80°29'01"E | 1 | Least quality | 1 | true |
| LEQL3 | 7°55'09"N | 80°27'31"E | 1 | Least quality | 1 | true |
| LEQL4 | 7°46'52"N | 80°29'09"E | 1 | Least quality | 1 | true |
| LEQL5 | 7°49'54"N | 80°26'54"E | 2 | Low quality | 1 | false |
| LEQL6 | 7°52'26"N | 80°28'11"E | 1 | Least quality | 1 | true |
| LEQL7 | 7°44'04"N | 80°23'07"E | 1 | Least quality | 1 | true |
| LEQL8 | 7°46'27"N | 80°28'14"E | 1 | Least quality | 1 | true |
| LEQL9 | 7°58'54"N | 80°25'37"E | 1 | Least quality | 1 | true |
| LEQL10 | 7°43'37"N | 80°24'19"E | 1 | Least quality | 1 | true |
| LQL1 | 7°51'13"N | 80°24'54"E | 2 | Low quality | 2 | true |
| LQL2 | 7°48'37"N | 80°27'21"E | 2 | Low quality | 2 | true |
| LQL3 | 7°47'44"N | 80°23'24"E | 1 | Least quality | 2 | false |
| LQL4 | 7°42'07"N | 80°30'35"E | 1 | Least quality | 2 | false |
| LQL5 | 7°55'54"N | 80°25'19"E | 2 | Low quality | 2 | true |
| LQL6 | 7°45'13"N | 80°30'47"E | 2 | Low quality | 2 | true |
| LQL7 | 7°47'28"N | 80°25'22"E | 2 | Low quality | 2 | true |
| LQL8 | 7°45'05"N | 80°27'47"E | 2 | Low quality | 2 | true |
| LQL9 | 7°42'45"N | 80°22'59"E | 2 | Low quality | 2 | true |
| LQL10 | 7°42'21"N | 80°20'45"E | 1 | Least quality | 2 | false |
| MQL1 | 7°41'29"N | 80°22'06"E | 3 | Moderate quality | 3 | true |
| MQL2 | 7°42'42"N | 80°30'15"E | 3 | Moderate quality | 3 | true |
| MQL3 | 7°46'13"N | 80°26'07"E | 2 | Low quality | 3 | false |
| MQL4 | 7°48'59"N | 80°24'20"E | 3 | Moderate quality | 3 | true |
| MQL5 | 7°45'57"N | 80°23'42"E | 3 | Moderate quality | 3 | true |
| MQL6 | 7°43'24"N | 80°26'56"E | 3 | Moderate quality | 3 | true |
| MQL7 | 7°54'06"N | 80°25'35"E | 3 | Moderate quality | 3 | true |
| MQL8 | 7°47'12"N | 80°21'49"E | 3 | Moderate quality | 3 | true |
| MQL9 | 7°43'25"N | 80°20'53"E | 3 | Moderate quality | 3 | true |
| MQL10 | 7°41'29"N | 80°22'06"E | 3 | Moderate quality | 3 | true |
| HQL1 | 7°45'50"N | 80°22'12"E | 2 | Low quality | 4 | false |
| HQL2 | 7°46'20"N | 80°23'17"E | 2 | Low quality | 4 | false |
| HQL3 | 7°53'23"N | 80°23'54"E | 4 | High quality | 4 | true |
| HQL4 | 7°48'23"N | 80°22'00"E | 4 | High quality | 4 | true |
| HQL5 | 7°42'09"N | 80°24'46"E | 4 | High quality | 4 | true |
| HQL6 | 7°52'59"N | 80°23’27"E | 4 | High quality | 4 | true |
| HQL7 | 7°49'50"N | 80°22'45"E | 4 | High quality | 4 | true |
| HQL8 | 7°42'16"N | 80°21'59"E | 4 | High quality | 4 | true |
| HQL9 | 7°49'50"N | 80°22'45"E | 3 | Moderate quality | 4 | false |
| HQL10 | 7°48'52"N | 80°23'53"E | 4 | High quality | 4 | true |
